# Room temperature self-assembled growth of vertically aligned columnar copper oxide nanocomposite thin films on unmatched substrates

**DOI:** 10.1038/s41598-017-10540-6

**Published:** 2017-09-11

**Authors:** Y. Wang, J. Ghanbaja, S. Bruyère, F. Soldera, D. Horwat, F. Mücklich, J. F. Pierson

**Affiliations:** 1 Institut Jean Lamour, UMR 7198-CNRS, Université de Lorraine, Nancy, F-54000 France; 2 State Key Laboratory Cultivation Base for Nonmetal Composites and Functional Materials, Southwest University of Science and Technology, Mianyang, 621010 China; 3 Department for Materials Science, Functional Materials, Saarland University, Saarbrücken, D-66123 Germany

## Abstract

In this work, we report the self-assembled growth of vertically aligned columnar Cu_2_O + Cu_4_O_3_ nanocomposite thin films on glass and silicon substrates by reactive sputtering at room temperature. Microstructure analyses show that each phase in nanocomposite films has the columnar growth along the whole thickness, while each column exhibits the single phase characteristics. The local epitaxial growth behavior of Cu_2_O is thought to be responsible for such an unusual microstructure. The intermediate oxygen flow rate between those required to synthesize single phase Cu_2_O and Cu_4_O_3_ films produces some Cu_2_O nuclei, and then the local epitaxial growth provides a strong driving force to promote Cu_2_O nuclei to grow sequentially, giving rise to Cu_2_O columns along the whole thickness. Lower resistivity has been observed in such kind of nanocomposite thin films than that in single phase thin films, which may be due to the interface coupling between Cu_2_O and Cu_4_O_3_ columns.

## Introduction

Nanocomposite thin films have attracted much attention due to their peculiar and tailorable properties in a variety of applications, such as controlling the optical properties^1, 2^, reducing the dielectric loss^3–5^, tuning the magnetic and electrical transport properties^6–12^, enhancing the electrochemical activity^13, 14^, and increasing the hardness^15, 16^. The novel architecture, interfacial interplay, and interaction or coupling between the constituents, are thought to be responsible for these peculiar properties and new functionalities. The nanocomposite thin films are typically divided into four types from the viewpoint of microstructure, including nanoparticles in matrix, lamellar multilayer, columns in matrix and vertically aligned nanocomposites^17–20^. Among them, vertically aligned nanocomposite, where all the phases have the columnar growth along the film thickness direction, is particularly appealing as its larger interfacial area and high availability of vertical strain control than other types^17, 18^.

In the past decade, tremendous progress has been made in nanocomposite thin films about designing interface-induced novel functionality. However, most of investigations on vertically aligned oxide nanocomposites are dedicated to epitaxial films (i.e., one perovskite phase with another phase) grown on matched single-crystal oxide substrates at high temperature by pulsed laser deposition^3, 5, 8, 9, 11, 13, 17, 18^. Such matched single crystal substrates are critical to promote the separated and independent columnar growth for different phases. Furthermore, the cost and the small dimension of the substrate, as well as the low scale from pulsed laser deposition, are not desirable for the large-area devices. Hence, to promote the application of vertically aligned nanocomposite thin films in different areas, it is attractive to deposit such films on unmatched substrates using a low-cost method. Magnetron sputtering is a standard manufacturing process associated with relatively low cost and easy fabrication of large-area films. Sputtered films with single phase usually exhibit the columnar microstructure^15, 21, 22^. However, this typical columnar structure vanishes with the addition of a second phase^15^. Therefore, it is challengeable to grow biphase composite thin films with vertically aligned columnar microstructures on unmatched substrates.

Binary copper oxides (Cu_2_O, Cu_4_O_3_ and CuO), as spontaneous p-type semiconductors, have been widely studied^23–28^. More recently, some surprising properties have been observed in the biphase copper oxide composite thin films. For instance, a lower resistivity has been observed in biphase sputtered Cu_2_O + Cu_4_O_3_ thin films than in the single phase Cu_2_O or Cu_4_O_3_^26^. In addition, the biphase Cu_2_O and Cu_4_O_3_ thin films can enhance the photovoltaic activity significantly in a binary copper oxide (Cu-O) light absorber^27^. However, the origin of these peculiar properties remains unknown.

In this work, we demonstrate the vertically aligned columnar microstructure of biphase Cu_2_O + Cu_4_O_3_ nanocomposite thin films grown by reactive magnetron sputtering at room temperature on unmatched glass or silicon substrates. Finally, the unusual electrical properties of biphase thin films are discussed.

## Results

**Figure 1:**
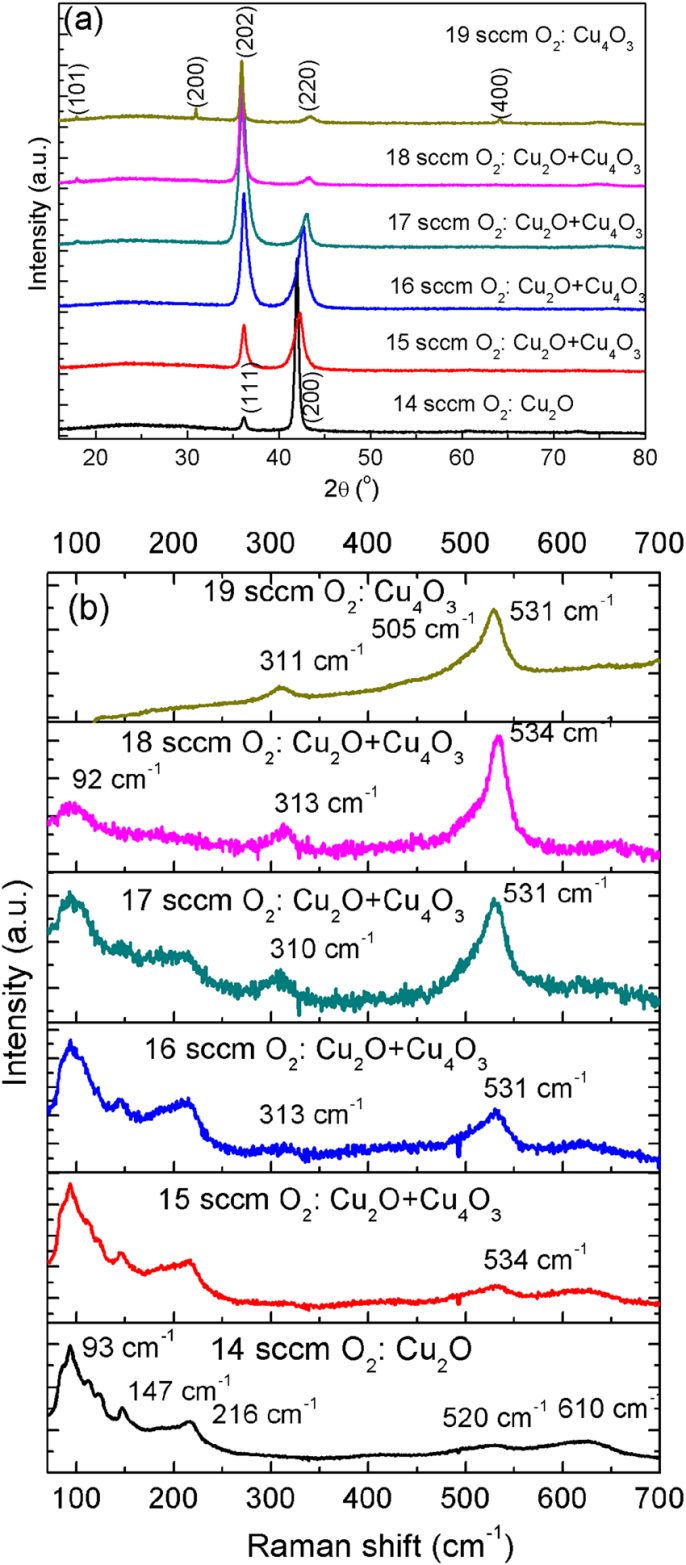
(**a**) X-ray diffractograms and (**b**) Raman spectra of copper oxide thin films deposited with different oxygen flow rates.

The diffractograms of copper oxide thin films deposited with different oxygen flow rates are presented in Fig. 1(a). Two main diffraction peaks are always observed at approx. 36° or 42° in these oxygen flow rates. The first peak may be due to the diffraction of Cu_2_O (111) planes or Cu_4_O_3_ (202) ones and the peak located close to 42° may be related to Cu_2_O (200) or Cu_4_O_3_ (220), as the *d* values in Cu_2_O and Cu_4_O_3_ are quite close in certain planes (see the supporting information). To obtain a more precise structural description of the films, micro-Raman spectrometry was used (Fig. 1(b)). The film deposited with 14 sccm oxygen shows a typical Raman spectrum of Cu_2_O, where the T_2g_ peak is observed close to 520 cm^−1^. The bands at 93, 147 and 216 cm^−1^ are related to defects, non-stoichiometry and resonant excitation in Cu_2_O^29^. A new band close to 531 cm^−1^ is evidenced when the oxygen flow rate is 15 sccm, which has been assigned to A_1g_ mode of Cu_4_O_3_^29, 30^. Its intensity increases with the increase of oxygen flow rate while other bands related to Cu_2_O decrease progressively. Hence, these Raman spectra clearly evidence that the films deposited with 15–18 sccm of oxygen are biphase composite Cu_2_O + Cu_4_O_3_ thin films, and that the fraction of Cu_4_O_3_ can be controlled by adjusting the oxygen flow rate.

**Figure 2:**
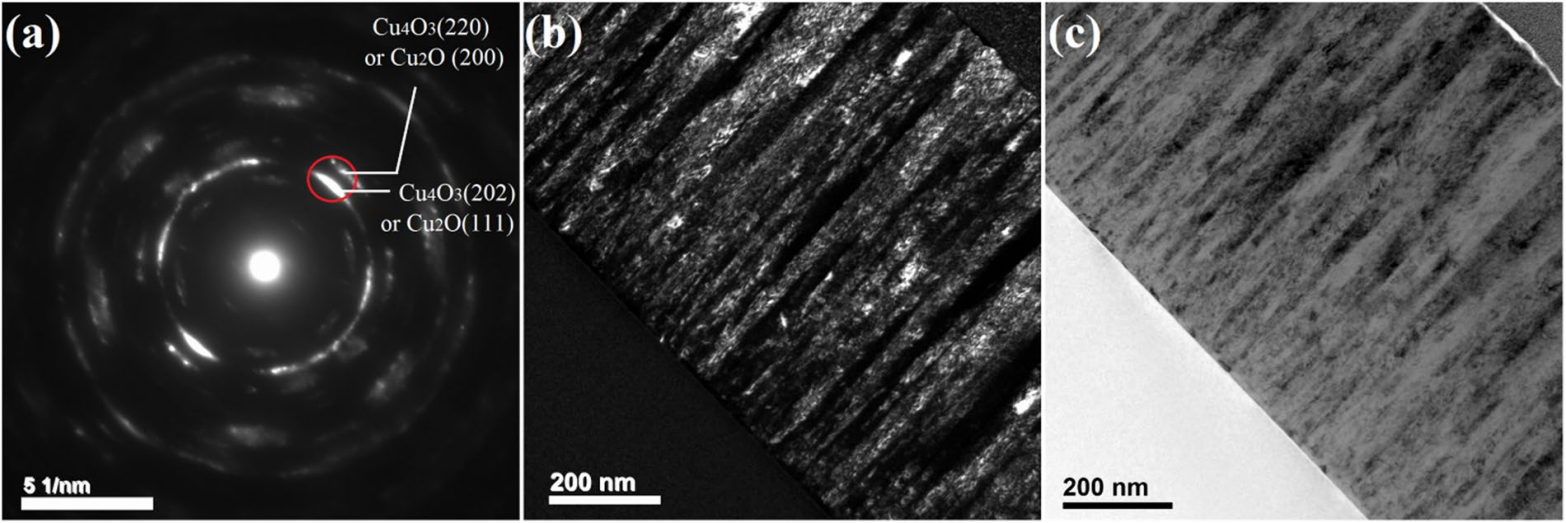
Cross-sectional TEM micrographs of biphase Cu_4_O_3_ and Cu_2_O thin film. (**a**) Electron diffraction pattern. The red circle represents the selected region for dark field image. (**b**) Dark field image. (**c**) Bright field image.

To study the microstructure of the biphase thin films, transmission electron microscopy (TEM) analyses were carried out in cross-section firstly. The cross-sectional TEM images of biphase Cu_4_O_3_ and Cu_2_O thin film deposited with 17 sccm O_2_ are shown in Fig. 2. Electron diffraction pattern on a large area is presented in Fig. 2(a), which can hardly distinguish Cu_2_O and Cu_4_O_3_ phases since their *d* values are close to each other (see the supporting information). Surprisingly, the dark and bright field images in Fig. 2(b,c) show notable columnar growth for this biphase film, and the columns start from the film/substrate interface to the top of the film, which is unusual in sputtered composite thin films. Such microstructure is quite similar with that in single phase Cu_2_O or Cu_4_O_3_ thin films^29^. However, the column width of about 20–40 nm near the top of this biphase film is much smaller than that of 30–70 nm in single phase Cu_4_O_3_ thin films^29^, indicating the existence of competing growth in this biphase thin film. Unfortunately, it is difficult to identify Cu_2_O and Cu_4_O_3_ phases from dark field image by choosing the corresponding diffraction spots, as the *d* values of main diffraction spots are too close (see Fig. 2(a)).

**Figure 3:**
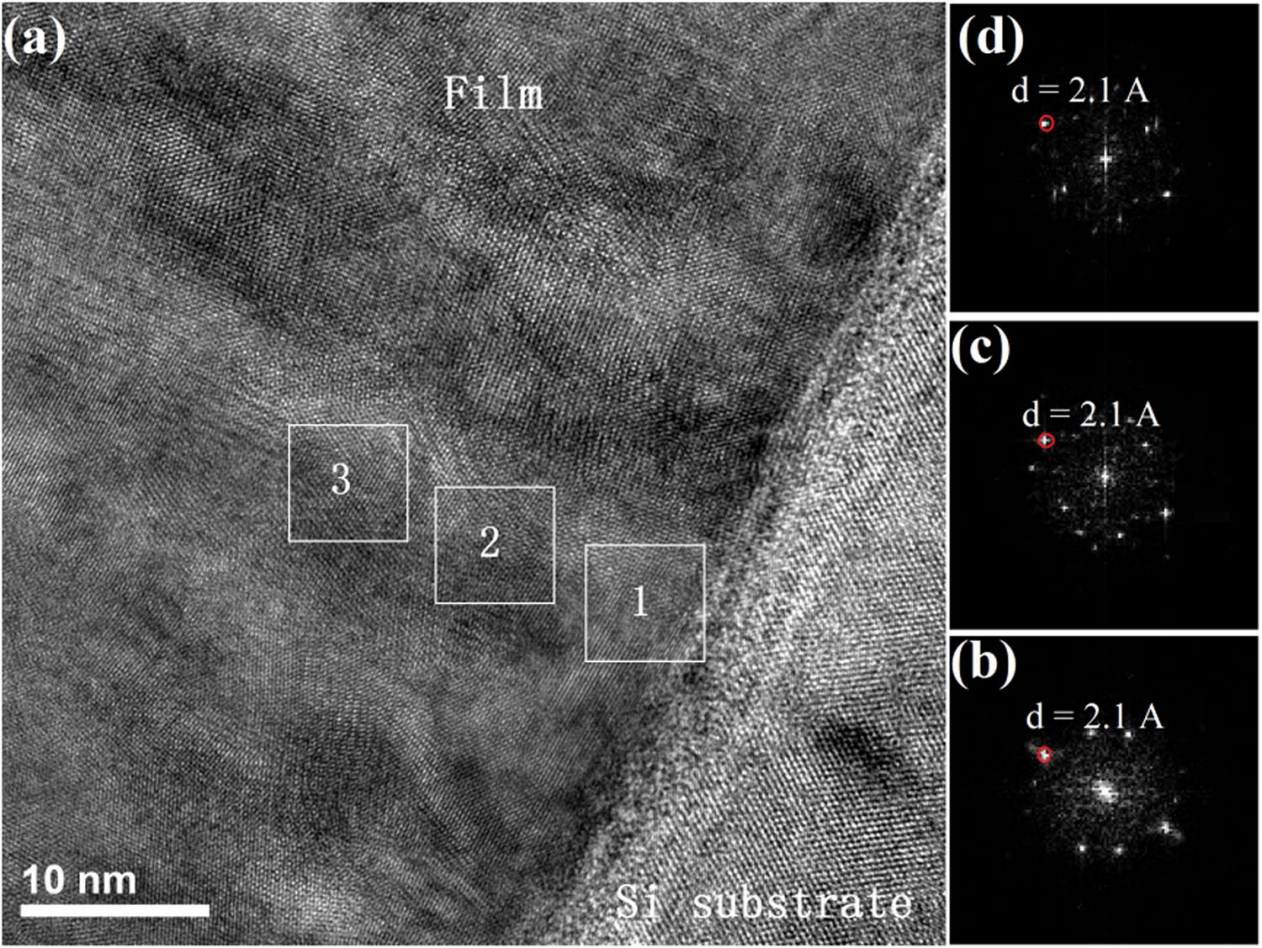
(**a**) HRTEM image of biphase thin film deposited with 17 sccm O_2_ at the initial growth region. Along the column growth direction, three square regions are marked as 1, 2 and 3. (**b**–**d**) Are the FFT patterns of square regions 1, 2 and 3 in (**a**), respectively. The red circles in (**b**–**d**) represent the diffraction spots along the column growth direction, with the *d* values of about 2.1 Å.

Furthermore, the microstructure at the initial growth region (close to the substrate) has been studied by high resolution TEM (HRTEM), as shown in Fig. 3. Even at the initial growth region, the biphase film still has the columnar microstructure, with the column width of about 10 nm. The fast Fourier transform (FFT) analyses along the column growth direction have been performed. Figure 3(b–d) show the FFT patterns of square regions named as 1, 2 and 3 in Fig. 3(a), respectively. It is clearly seen that *d* values of about 2.1 Å have always been observed along the column growth direction, as shown in Fig. 3(b–d). This *d* value of 2.1 Å could come from Cu_2_O (200) or Cu_4_O_3_ (220), as the information in these patterns is not sufficient to determine the phase structures. To be pointed out here, the poor FFT patterns in Fig. 3(b–d) are typical ones in polycrystalline thin films, which originates from the characteristics of small column width and fiber texture. The thickness of the TEM foil is estimated to be about 50–70 nm by low loss electron energy loss spectroscopy (EELS), much larger than the column width near the substrate, which indicates that there are several columns along the TEM thin foil thickness direction. Besides, the fiber texture observed in pure phase Cu_2_O and Cu_4_O_3_ thin films, may exist in this biphase thin film. Hence, several columns with some rotational degree of freedom around the fiber axis will result in poor diffraction spots. Whatever this diffraction spot belongs to Cu_2_O or Cu_4_O_3_, such analyses indicate that the columnar microstructure in the biphase thin film is formed at the beginning of the growth process, and the columns have almost the same growth orientation along the whole thin film thickness.

**Figure 4:**
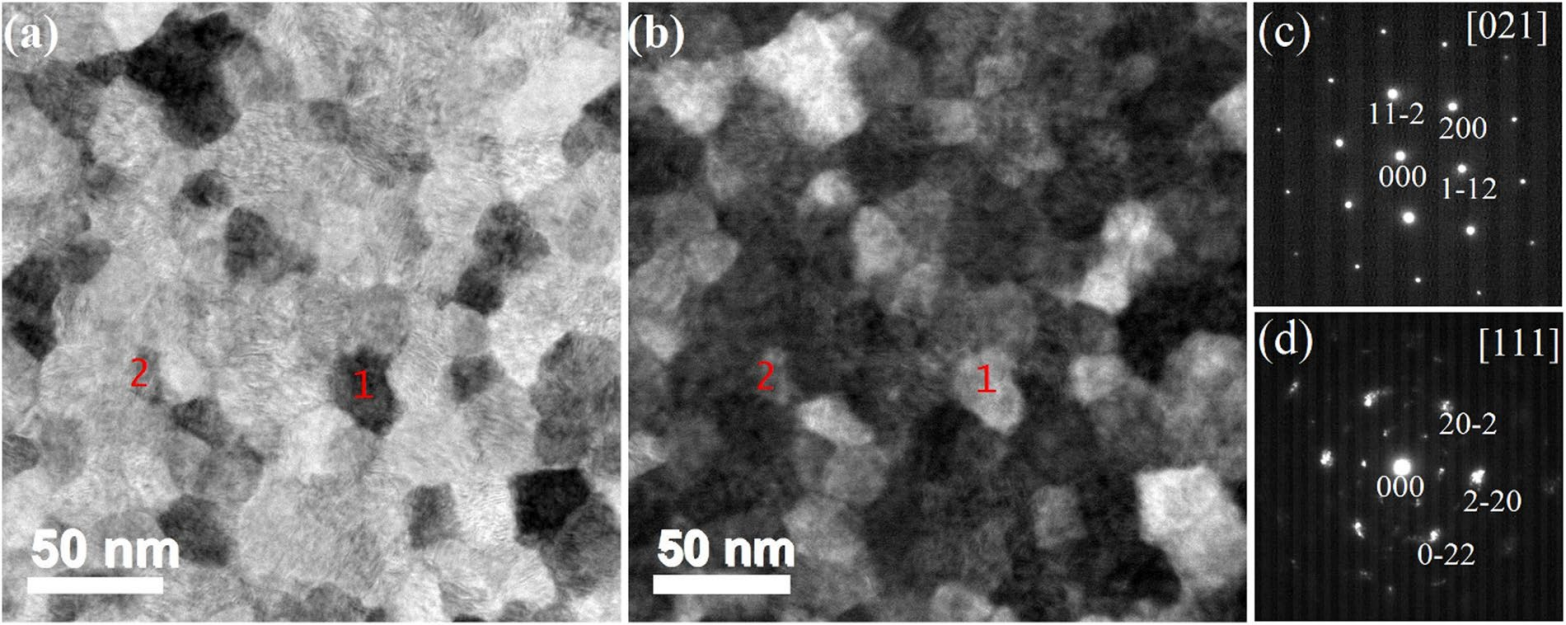
Top-view TEM micrographs of biphase Cu_4_O_3_ and Cu_2_O thin film with 17 sccm O_2_. (**a**,**b**) are bright and dark field images, respectively. Two grains referred as # 1 and # 2, have been marked. (**c**,**d**) are micro-diffraction patterns of grain #1 and #2, respectively.

To capture the microstructure of the biphase thin film unambiguously, TEM investigations have also been performed on foils prepared parallel to the film surface, i. e. from the top-view of the specimen. Electron diffraction patterns have been recorded from many grains, and typical patterns are shown in Fig. 4. Figure 4(a) is the bright field image and Fig. 4(b) is the corresponding dark field image, in which the estimated grain size of about 20–40 nm is consistent with the column width in cross-sectional micrographs. In Fig. 4(a,b), grains referred as # 1 and # 2 have been marked. The micro-diffraction patterns of grains #1 (see Fig. 4(c)) exhibits the single crystal diffraction characteristic of Cu_4_O_3_, clearly demonstrating this grain is single phase Cu_4_O_3_. The diffraction pattern of grain #2 is displayed in Fig. 4(d), which shows the characteristic of Cu_2_O as the main diffraction spots can be only indexed by cubic crystal structure, rather than tetragonal structure. As shown in Fig. 4(d), a little vestige of diffraction ring has also been observed, which could be due to the small grain size. Then, the convergent beam electron diffraction (CBED) has been performed using another microscope (Philips CM200). The CBED pattern clearly reveals the single phase of Cu_2_O grain as the pattern shows notable single crystal characteristic (see the supporting information). The CBED pattern of Cu_4_O_3_ also confirms its pure phase for every grain (see the supporting information).

**Figure 5:**
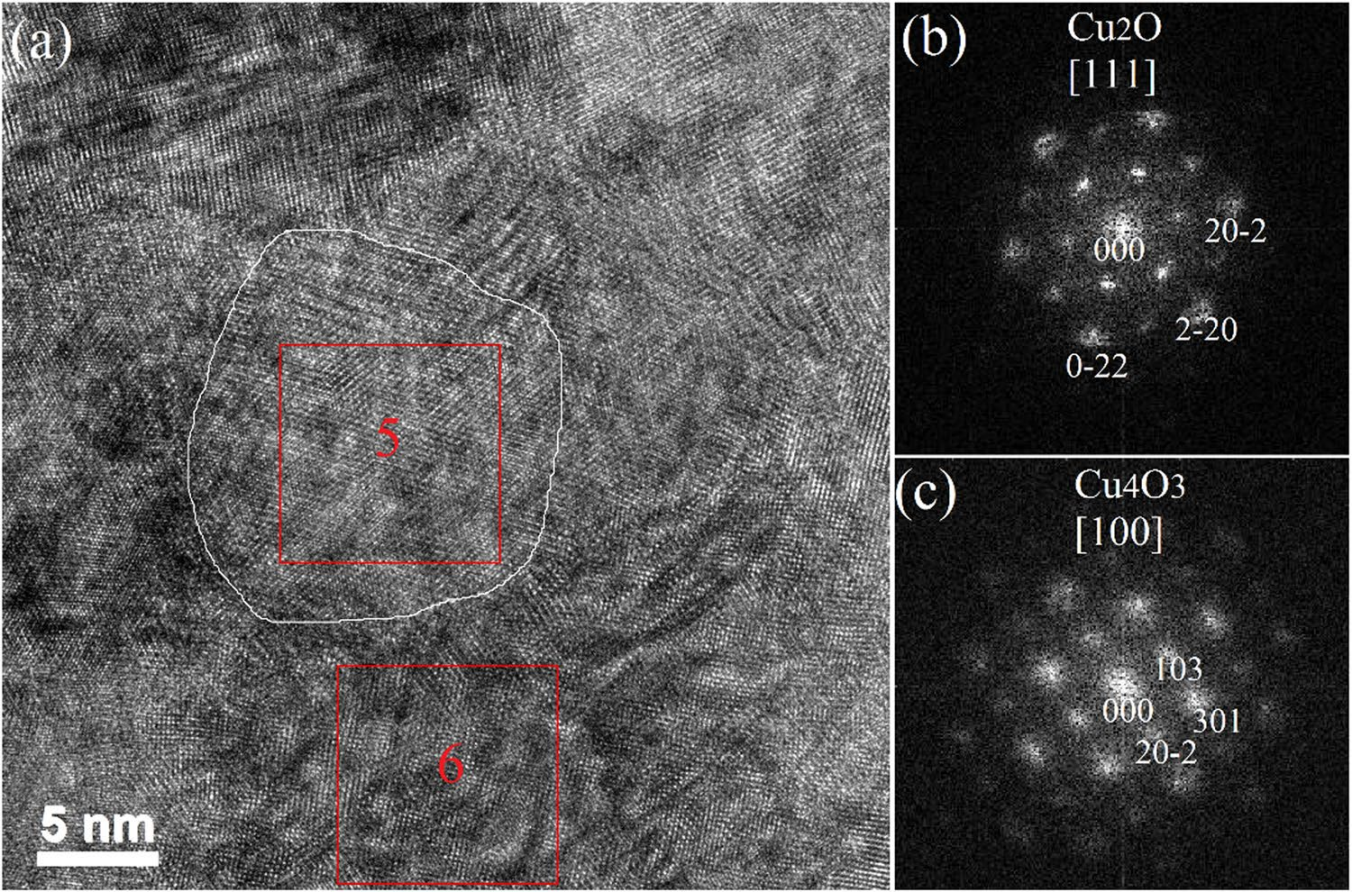
(**a**) Top-view HRTEM micrograph of biphase Cu_4_O_3_ and Cu_2_O thin film with 17 sccm O_2_. Two grains referred as #5 and #6, have been marked. The white line roughly describes the grain boundaries of #5. The red square frames represent the selected regions for FFT analysis. (**b**,**c**) are the FFT patterns of grains #5 and #6, respectively.

Furthermore, the single phase characteristic of different grains has also been studied by HRTEM. As shown in Fig. 5(a), two grains labelled as #5 and #6 have been chosen to perform FFT analyses. The FFT pattern of #5 (see Fig. 5(c)) demonstrates this grain to be single phase cubic Cu_2_O, as tetragonal structure does not exhibit the six-fold symmetry. Figure 5(d) is the FFT pattern of grain #6, which is well indexed as tetragonal Cu_4_O_3_, indicating its single phase characteristic. Thus, the HRTEM analyses also verify that both Cu_2_O and Cu_4_O_3_ grains are pure phase.

**Figure 6:**
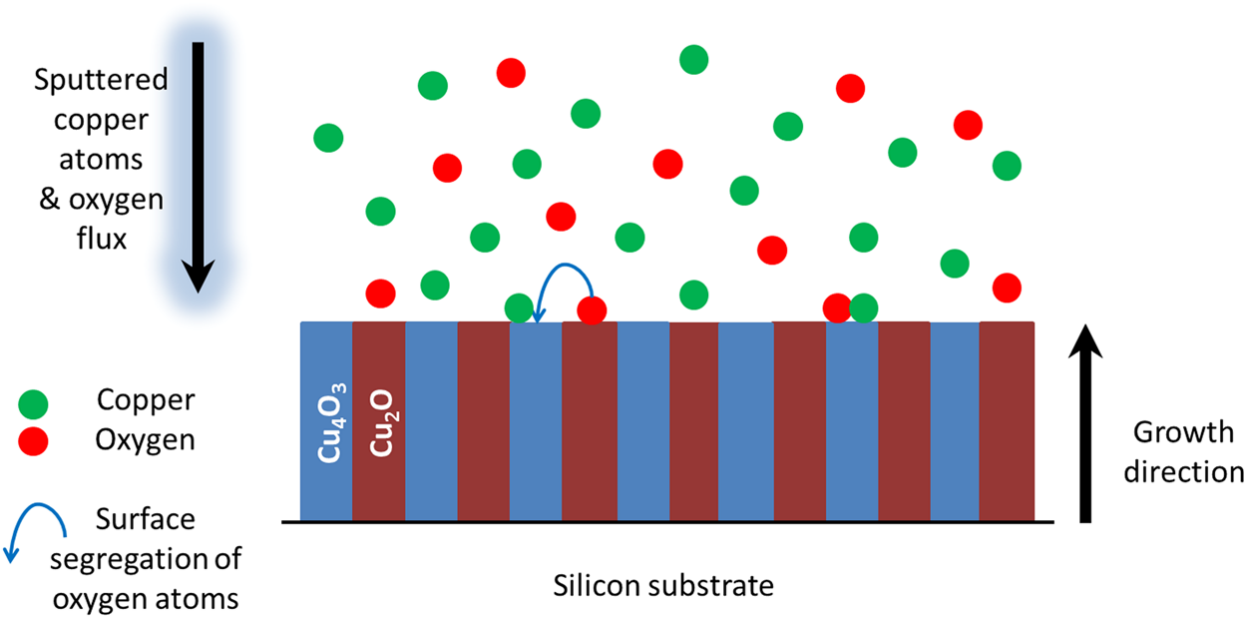
Schematic microstructure and the formation mechanism of the biphase Cu_4_O_3_ and Cu_2_O thin film. For simplicity, we show an ordered arrangement of phase.

The above TEM micrographs from cross-section and top-view indicate an unusual microstructure in biphase Cu_4_O_3_ and Cu_2_O thin films where the two phases grow independently in columnar shape. It is worth noting that this kind of microstructure has clearly evidenced in biphase Cu_4_O_3_ and Cu_2_O thin films with different oxygen flow rates of 16, 17 and 18 sccm. Such a microstructure is significantly different from the traditional concept that one phase is embedded into the second one that acts as matrix. Hence, the schematic microstructure of this biphase thin film is depicted in Fig. 6; for simplicity, we show an ordered arrangement of phases. As shown in Fig. 6, both phases just grow separately and independently with the columnar microstructure along the whole film thickness. This kind of unusual growth can be understood from the viewpoint of Cu_2_O local epitaxial growth (LEG) behavior previously reported^31^. In reactively sputtered growth of Cu_2_O thin films, the Cu_2_O seed layer has a strong driving force to promote the subsequent growth with the same growth orientation, independently of the deposition conditions^31^. Therefore, in this biphase thin film, the growth process can be assumed as follows: (1) due to intermediate oxygen flow rate between those required to grow single phase Cu_2_O and Cu_4_O_3_, some Cu_2_O nuclei are formed; (2) the strong driving force resulting from the local epitaxial growth induces a selective formation of Cu_2_O on the nuclei with the same structure; (3) the local decrease of the oxygen concentration induces a segregation of oxygen adatoms towards columns with higher oxygen concentration that crystallizes in the Cu_4_O_3_ structure. Consequently, Cu_4_O_3_ and Cu_2_O phases with columnar structures grow independently.

**Figure 7:**
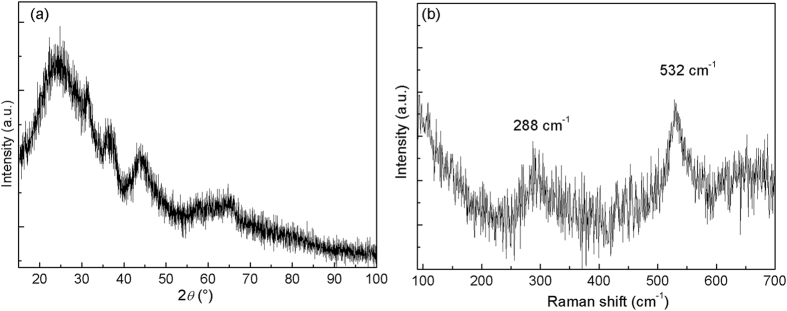
(**a**) X-ray diffractogram and (**b**) Raman spectrum of biphase Cu_4_O_3_ and CuO thin films.

**Figure 8:**
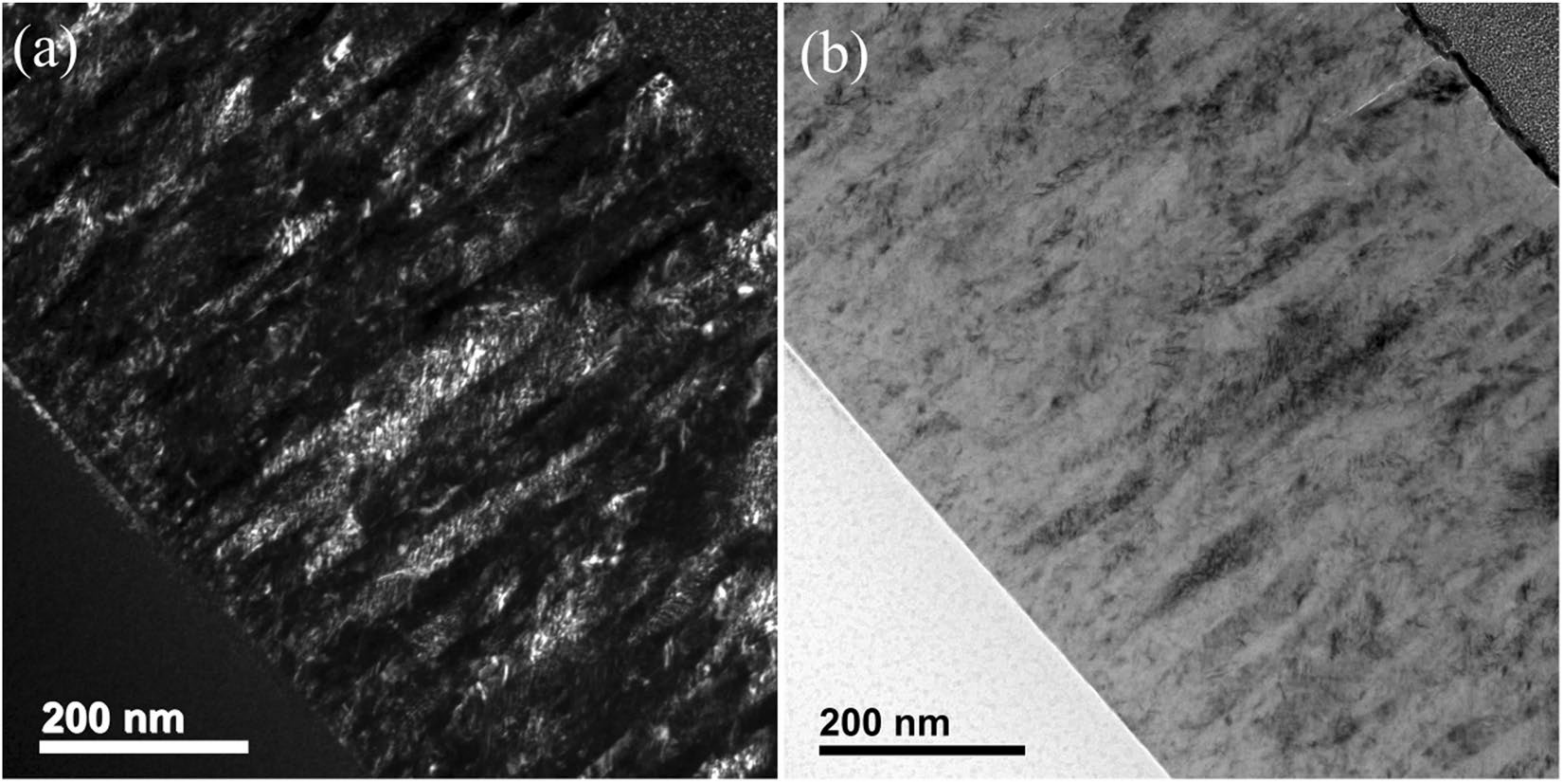
Cross-sectional TEM micrographs of biphase Cu_4_O_3_ and CuO thin film. (**a**) Dark field image. (**b**) Bright field image.

As previously reported, the oxygen flow rate allows to tune the phase structure of copper oxide films^29^. The increase of the oxygen flow rate induces the deposition of Cu_2_O, Cu_4_O_3_ and CuO. Moreover, between these single phases, biphase Cu_2_O + Cu_4_O_3_ and Cu_4_O_3_ + CuO films can also be synthesized. The structure and the microstructure of Cu_4_O_3_ + CuO films have also been studied by XRD, Raman and TEM. Films deposited with 21 sccm O_2_ are X-ray amorphous (Fig. 7(a)), but Raman analyses clearly evidence the existence of Cu_4_O_3_ A_1g_ mode close to 531 cm^−1^ and CuO A_g_ mode at about 288 cm^−1^ (Fig. 7(b))^29, 30^. Compared to Cu_2_O + Cu_4_O_3_ biphase films, the Cu_4_O_3_ + CuO ones show notably different microstructure. From the cross-sectional TEM images, the columnar growth in biphase Cu_4_O_3_ + CuO thin film is not clear (see Fig. 8). Moreover, the top-view electron diffraction patterns can hardly identify the single phase features of grains. Hence, the vertically aligned columnar growth mechanism is not encountered in the biphase Cu_4_O_3_ + CuO film anymore. This result can also be explained by taking the LEG effect into account. Indeed, the texture of CuO films is mainly governed by the oxygen partial pressure^29^. Thus, a local change of the oxygen concentration induces a change of the CuO preferred orientation that comes with a nucleation of a new grain without structural relationship with the previous one. Consequently, there is no LEG behavior in this oxide. In the case of Cu_4_O_3_ phase_,_ the [101] orientation deposited at 0.5 Pa does not allow the LEG effect. Considering the occurrence of LEG effect in Cu_2_O thin films, the vertically aligned columnar growth mechanism in biphase Cu_2_O + Cu_4_O_3_ films can be well described. On the other hand, this growth mechanism is not encountered in biphase Cu_4_O_3_ + CuO ones (no LEG effect in these two phases within the present growth conditions). Within this discussion, it is believed that this vertically aligned columnar growth observed in biphase Cu_2_O + Cu_4_O_3_ thin films can also be extended to other materials with certain requirements summarized as below:

- The system has to contain at least two stable or metastable phases,
- Each phase has to be deposited in crystalline form within the deposition conditions,
- The growth rate of each phase has to be similar. Within the Cu-O system, the growth rate of Cu_2_O is close to that of Cu_4_O_3_, while that of CuO is relatively low (poisoning effect of the target)^29, 32^,
- At least one phase should be grown independently with a local epitaxial growth mechanism,
- The chemical compositions of the phases must be close, in order to allow the segregation of one adsorbed element on the growing surface.

**Figure 9:**
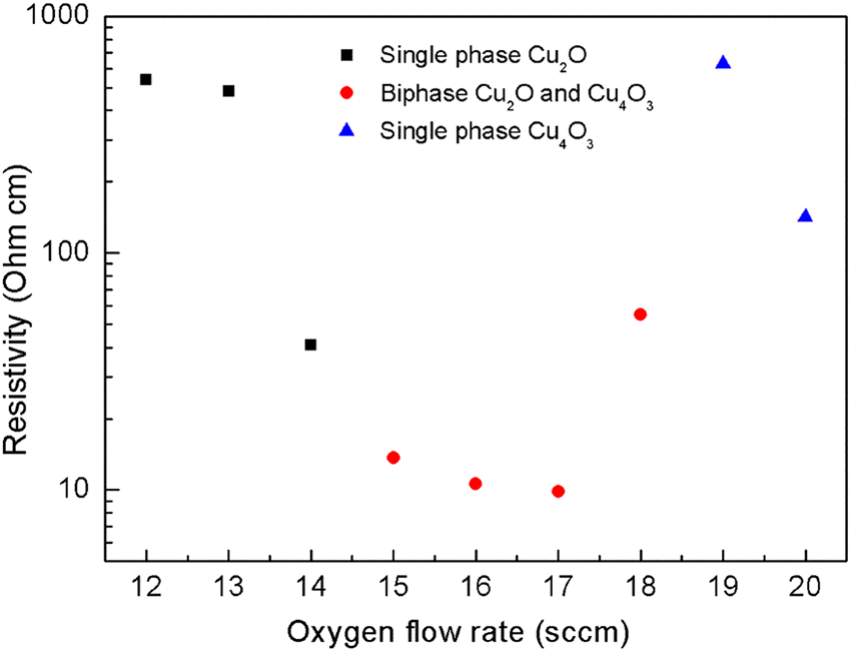
Resistivity of copper oxide thin films as a function of oxygen flow rate.

The room temperature resistivity of copper oxide thin films as a function of oxygen flow rate is depicted in Fig. 9, which clearly reveals that the biphase thin film has lower resistivity than single phase films. This result is in agreement with that reported by Meyer *et al*.^26^. Since these thin films are deposited at room temperature and the mobility is extremely low, it is difficult to determine the carrier concentration by Hall effect measurements. For the single phase Cu_2_O or Cu_4_O_3_ thin films, the room temperature resistivity decreases with the increase of oxygen flow rate (see Fig. 9), which could be qualitatively understood from the defect mechanism. Taking Cu_2_O as an example, copper vacancy (${{\rm{V}}}_{\mathrm{Cu}}^{^{\prime} }$) is the predominant defects to produce the hole carriers, while the formation energy of copper vacancy decreases in the oxygen rich conditions (higher oxygen flow rate)^33–35^. Then the lower resistivity of single phase Cu_2_O thin films with higher oxygen flow rate can be interpreted from its larger carrier concentration due to the reduction of copper vacancy formation energy. In the case of biphase Cu_2_O and Cu_4_O_3_ thin film, the oxygen flow rate is higher than that required to synthesis the single phase Cu_2_O, thus the Cu_2_O columns may have higher carrier concentration. In contrast, the Cu_4_O_3_ columns may have lower carrier concentration as the oxygen sub-stoichiometry. Consequently, the columns with different carrier concentration (high carrier concentration and low carrier concentration) arrange randomly, and their interface coupling may play a role in the establishment of lower resistivity. Further investigations are required to clarify this unusual phenomenon.

## Conclusions

An unusual microstructure has been observed in biphase Cu_2_O + Cu_4_O_3_ nanocomposite thin films grown on glass and silicon substrates by reactive sputtering at room temperature, where two phases grow separately and independently with vertically aligned columnar microstructure along the whole film thickness. Such a microstructure may relate to the local epitaxial growth of Cu_2_O. The intermediate oxygen flow rate between those required to grow pure phase Cu_2_O and Cu_4_O_3_ thin films produce some Cu_2_O nuclei, and then the strong driving force resulting from the local epitaxial growth induces a selective formation of Cu_2_O on the nuclei with the same structure, giving rise to this kind of unusual vertically aligned columnar microstructure on unmatched substrates. Such peculiar microstructure can also be extended to other materials with certain requirements. This vertically aligned columnar Cu_2_O + Cu_4_O_3_ nanocomposite thin film exhibits much lower resistivity than single phase thin films, which may be due to the strong interface coupling between Cu_2_O and Cu_4_O_3_ columns.

## Methods

### Film growth

Copper oxide thin films were deposited on glass substrates (microscopy slides) and (100) silicon single crystal substrates by reactive pulsed-DC magnetron sputtering in Ar-O_2_ reactive mixtures. The amorphous SiO_2_ layer on silicon single crystal substrate was not removed, giving rise to the same characteristics of silicon and glass substrates. Thus, the substrates had no effect on the growth orientation and phase structure of thin films. No intentional heating was applied to the substrates, and the deposition temperature was close to room temperature. The argon flow rate was fixed at 25 sccm, while the oxygen flow rate varied in the range of 12–21 sccm with a step of 1 sccm. The accuracy of gas flow controller (Air Liquide) is +/− 0.1 sccm in this work. A pulsed-DC supply (Pinnacle + Advanced Energy) was used to sputter the copper target (50 mm diameter and 3 mm thick with a purity of 99.99%). The current applied to target was fixed to 0.3 A, the frequency and the off-time were 50 kHz and 4 µs, respectively. The distance between the substrate and the target was fixed at 60 mm.

### Characterizations

X-ray diffraction (XRD, Brucker D8 Advance with CuK_α1_ radiation (*λ* = 0.15406 nm) in Bragg Brentano configuration) and micro-Raman spectrometry (Horiba LabRAM HR using a 532 nm laser) were employed together to identify the phase structures. Transmission electron microscopy (TEM) investigations were performed by a JEOL ARM 200-Cold FEG (point resolution 0.19 nm) fitted with a GIF Quantum ER. For this purpose, the TEM cross-section and top-view specimens of composite thin films deposited on silicon substrates were prepared in a focused ion beam (FIB)-scanning electron microscope (SEM) dual beam system (FEI Helios 600) using the ‘*in situ*’ lift-out technique. Final thinning was done with low voltage milling (5 kV) to reduce any possible preparation artifacts. The convergent beam electron diffraction (CBED) analyses were done by another TEM (Philips CM200). Besides, the top-view microstructure was also studied by TEM specimens prepared by diamond tip cleave. Electrical resistivity measurements were performed at room temperature using the four-point probe method.

## Electronic supplementary material

supplementary info
