# Supplementary material for: Room temperature self-assembled growth of vertically aligned columnar copper oxide nanocomposite thin films on unmatched substrates
Source: Sci Rep. 2017 Sep 11;7:11122. doi: 10.1038/s41598-017-10540-6 (PMC5594013; doi:10.1038/s41598-017-10540-6)
Supplement: Supplementary file 1 — supplementary info [file 41598_2017_10540_MOESM1_ESM.pdf]

Supporting information

Room temperature self-assembled growth of vertically aligned columnar copper oxide nanocomposite thin films on unmatched substrates

Y. Wang[1,2], J. Ghanbaja[1], S. Bruyère [1], F. Soldera[3], D. Horwat[1], F. Mücklich[3], J.F. Pierson[1,*]

[1] Institut Jean Lamour, UMR 7198-CNRS, Université de Lorraine, Nancy F-54000, France.

[2] State Key Laboratory Cultivation Base for Nonmetal Composites and Functional Materials, Southwest University of Science and Technology, Mianyang 621010, China.

[3] Department for Materials Science, Functional Materials, Saarland University, Saarbrücken D-66123, Germany.

[*]Corresponding author: J. F. Pierson Email: jean-francois.pierson@univ-lorraine.fr

Tel : +33 (0)3 72 74 25 99

1. Special interplanar distances in Cu_2O and Cu_4O_3

Referring to the PDF cards of Cu_2O [JCPDS 04-007-9767] and Cu_4O_3 [JCPDS 04-007-2184], the distance of (111) planes in Cu_2O is quit close to those of (202) and (004) planes in Cu_4O_3, while the *d* value of Cu_2O (200) is also close to that of Cu_4O_3 (220), as shown in Table S1. This implies that it is a hard task to identify the phase structures of Cu_2O and Cu_4O_3 by X-ray diffraction, especially for the polycrystalline thin films, as the stress, defects, or stoichiometry may shift the diffraction peaks. Besides, the determining of phase structures in polycrystalline thin films by electron diffraction in transmission electron microscopy could be also very difficult.

Here it should be mentioned that Cu_4O_3 {001} may be polar surfaces with high surface energy. Along the <001> direction, Cu_4O_3 consists of alternative Cu and O atomic planes (see Fig. S1), which may give rise to a net surface charge and an electric dipole moment (infinite surface energy) in the repeat unit perpendicular to the {001} surfaces. Hence, it will be very difficult to grow Cu_4O_3 thin films with <001> growth orientation. Moreover, the pole figure measurements for single phase Cu_4O_3 thin film deposited with 19 sccm O_2 flow rate confirms that the X-ray diffraction peak at about 36° is mainly composed of (202) plane, rather than (004) plane, as the theoretical angle between (202) and (004) planes agrees well with the experimental χ value. Therefore, it is believed that the contribution of (004) planes to diffraction is very limit and will not be taken into consideration in this work.

Table S1 Special interplanar distances of Cu_2O and Cu_4O_3

Cu_2O	Cu_4O_3
$2\theta(111) = 36.428^o$<br>$d(111) = 2.464$ Å	$2\theta(202) = 35.654^o$<br>$d\,(202) = 2.516$ Å<br>$2\theta(004) = 36.146^o$<br>$d\,(004) = 2.483$ Å
$d(200) = 2.134$ Å	$d\,(220) = 2.064$ Å

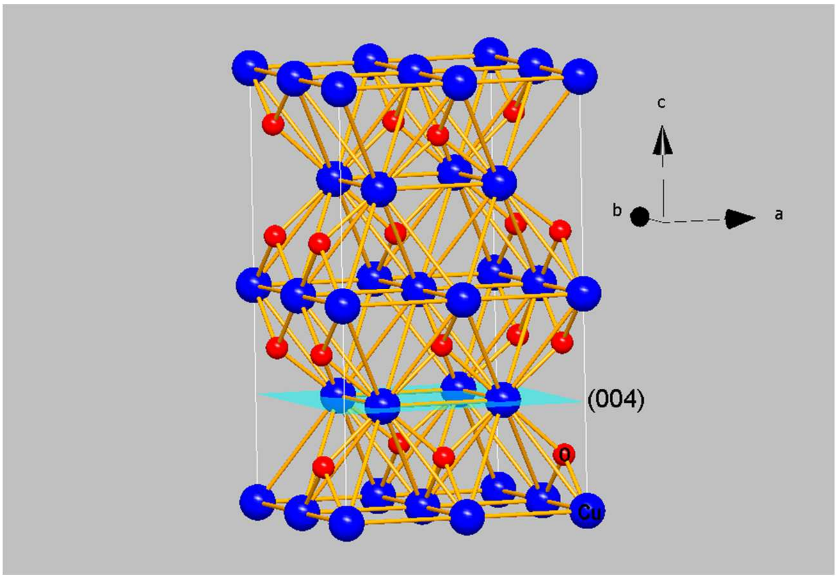

Fig. S1 Crystal structure of Cu_4O_3

2. CBED analyses of grains

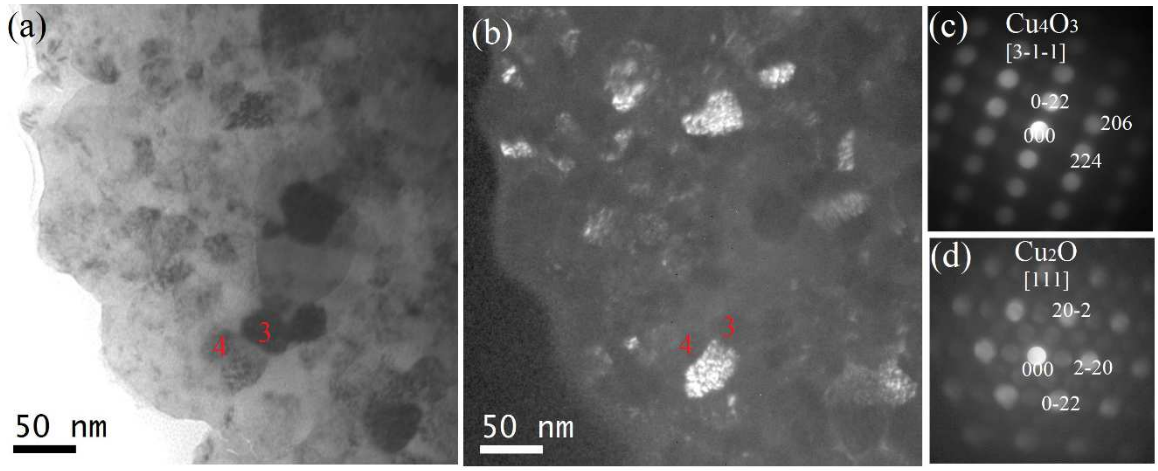

Fig. S2 Top-view TEM micrographies of biphase Cu_4O_3 and Cu_2O thin film with 17 sccm O_2. (a) and (b) are bright and dark field images, respectively. (c) and (d) are CBED patterns of grain #3 and #4 labeled in (a) and (b), respectively.

The convergent beam electron diffraction (CBED) analyses on top-view specimens have been performed by the microscopy of Philips CM200. Fig. S2(a) and (b) are the bright and dark field images, in which two grains have been marked as #3 and #4. The

CBED patterns of grains #3 and #4 are displayed in Fig. S2(c) and (d), respectively, which can be well indexed by cubic Cu_2O and tetragonal Cu_4O_3 with single crystal characteristic. Therefore, these CBED analyses reveal that every grain possesses the single phase structure, rather than the mixing of two phases.
